# Tautomerization of H^+^KPGG: Entropic Consequences of Strong Hydrogen-Bond Networks in Peptides

**DOI:** 10.1021/acs.jpca.3c03744

**Published:** 2023-07-25

**Authors:** Daniel Beckett, Tarick J. El-Baba, Zhichao Zhang, David E. Clemmer, Krishnan Raghavachari

**Affiliations:** † Department of Chemistry, Chicago Center for Theoretical Chemistry, The University of Chicago, Chicago, Illinois 60637, United States; ‡ Department of Chemistry, Indiana University, Bloomington, Indiana 47405, United States

## Abstract

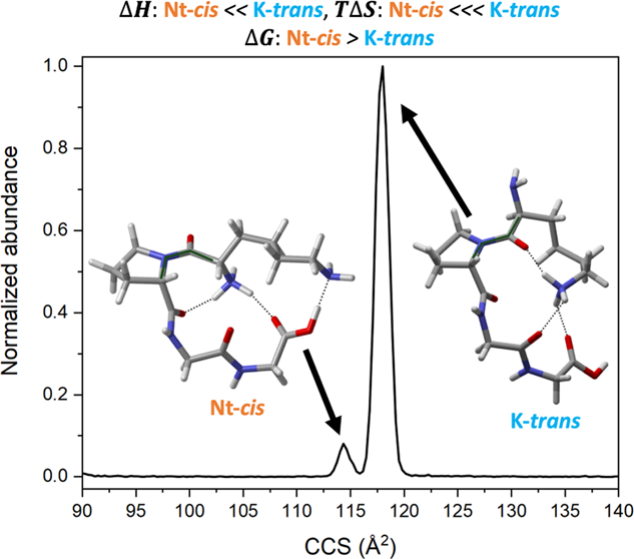

Ion mobility spectrometry-mass spectrometry and quantum chemical calculations are used to determine the structures and stabilities of the singly protonated peptide H^+^KPGG. The two peaks making up the IMS distribution are shown to be tautomers differing by the location of the extra proton on either the lysine side chain or the N-terminus. The lysine-protonated tautomer is strongly preferred entropically while being disfavored in terms of the electronic energy and enthalpy. This relationship is shown, through comparison of all low-lying conformers of both tautomers, to be related to the strong hydrogen-bond network of the N-terminally protonated tautomer. A general relationship is demonstrated wherein stronger cross-peptide hydrogen-bond networks result in entropically disfavored conformers. Further effects of the H^+^KPGG hydrogen-bond network are probed by computationally examining singly and doubly methylated analogues. These results demonstrate the importance of the entropic consequences of hydrogen bonds to peptide stability as well as techniques for perturbing the hydrogen-bond network and folding preferences of peptides via minimal chemical modification.

## 1 Introduction

Lysine (K, Lys) has the distinction of possessing the second highest gas-phase basicity of the 20 proteinogenic α-amino acids, behind arginine, due to the aliphatic side chain leading to an amino group creating a much more basic site than the N-terminus.^1^ Along these lines, a recent mutagenesis study on green fluorescent protein found that substitution of surface lysine residues with arginine led to stronger interactions (including salt-bridge interactions). However, despite the increased strength of arginine interactions, the specific strength of hydrogen bonds formed by the lysine side chain was discovered to guide the folding of the protein and the folding rate significantly decreased upon substitution.^2^ Furthermore, the specificity allowed by the lysine side chain has been linked to the regulation of calcium in mammals, such as in preventing arterial calcification in rats with chronic kidney disease,^3^ as well as to the production of the mitochondrial shuttle molecule carnitine.^4^

One major motivation in studying gas-phase, lysine-containing peptides is the large change in p*K*a observed in catalytic lysine residues buried deep in the hydrophobic core of proteins, a position seen to reduce lysine p*K*a to nearly half its surface value.^5^ The gas-phase distribution of a multiresidue peptide reflects the buried protein microenvironment wherein lysine engages in a high number of hydrogen bonds on average.^6^ Previously we have reported ion mobility spectrometry coupled with mass spectrometry (IMS-MS) of singly protonated GPGG to calibrate and test quantum chemical methods for the elucidation of peptide conformational distributions. Furthermore, we used our calibrated methodology along with the quantum theory of atoms in molecules (QTAIM) to probe the hydrogen-bond network of structurally similar singly protonated hairpin peptides (XPGG, X = D, E, N, Q) and understand how slight changes to hydrogen-bond networks affect electronic energies.^7,8^ These previous studies built up a theoretical framework we can take advantage of for the study of lysine. In line with this framework, we report the IMS spectrum of gas-phase H^+^KPGG, the simplest lysine-containing hairpin allowing intramolecular hydrogen-bonding interactions not taken into account when studying isolated lysine, as has been done with high-level quantum chemical calculations in the past.^1,9−11^

While gas-phase lysine calculations have found the protonation at the N-terminus to be significantly disfavored compared to protonation at the side-chain amine,^1,9−11^ the study of a lysine-containing hairpin lends insight into the reported p*K*a shift and whether this affects the protonation site. Additionally, methylated lysine acts as a key intermediate in carnitine biosynthesis^12,13^ and is also used in the experimental tagging of epigenetic markers in histones.^14^ Studies on methylated lysine are sparse, with two infrared (IR) action spectroscopy studies finding singly methylated lysine to possess a stronger proton affinity than unmethylated lysine and formation of a salt bridge when coupled with a metal cation.^15,16^ However, to the best of our knowledge, computational studies of methylated lysine in a hairpin peptide have not been reported and potential interplay between methylation and the surface/buried p*K*a shift should be explored, specifically with respect to the location of the excess proton.

This report follows a theoretical investigation of the experimental ion mobility spectrum of H^+^KPGG. First, we explore possible structures that are consistent with both experiment and theory using the experimental collision cross-section values and intensities as validation for computationally obtained conformers. The experimental spectrum is found to be composed of two tautomers, differing by location of the excess proton, and the location of the proton will be shown to be entropically driven. Following this, we will relate the entropic differences in low-lying conformers/tautomers of H^+^KPGG to differences in hydrogen-bond strength and investigate the hydrogen bonds within the most abundant conformers. Finally, we report a theoretical investigation on methylation of the lysine side chain. One methylation strongly favors the side-chain-protonated conformer, while addition of a second methyl group favors N-terminal protonation. These results unveil the role of entropy in tautomerization, the importance of hydrogen bonds in determining the relative entropic contributions of peptide conformers, and the effects of side-chain substitution on hydrogen bonding and tautomer preference.

## 2 Methods

### 2.1 Computational Details

Conformers for each peptide were generated in the same manner as in our previous studies on hairpin tetrapeptides.^7,8^ Starting structures for each peptide were built in a β-strand configuration with PCMODEL.^17^ To capture both tautomers, starting H^+^KPGG structures were built with the extra proton on either the lysine side-chain amino group or the N-terminal amino group and brought through the conformer generation process separately. Conformers were generated by stochastically rotating the rotatable bonds, quenching with the MMFF94 force field,^18^ and discarding structures outside of a 7 kcal/mol energy window. Generated conformers were optimized with the PM6 semiempirical method^19^ as implemented in Gaussian 16,^20^ and degenerate structures were discarded.

Conformers were further optimized, and frequencies obtained, with the CAM-B3LYP-D3BJ/6-311++G(d,p) level of theory,^21−28^ which was found to produce intensities closest to both experiment and CCSD(T)/CBS calculations in our previous work on H^+^GPGG.^7^ All structures were verified to be minima by frequency calculations, and thermochemical properties were obtained within the rigid rotor/harmonic oscillator approximation at 298.15 K and 1 atm.^20^ Frequencies were left unscaled, in line with our previous benchmarking,^7^ and no extra treatment of the frequencies (such as discarding low frequencies below a cutoff) was undergone due to the known importance of low frequencies in expressing the strength of hydrogen bonds in small-molecule dimers.^29,30^ This also aligns with the H^+^GPGG study wherein low-frequency modes were found to be essential to match the experimental distribution, not only in density functional calculations but also in CCSD(T)//MP2 conformational preferences.^7^

Collision cross sections were obtained via the trajectory method as implemented in MOBCAL^31^ and were averaged over 100 runs for each conformer, as they were in our previous work.^8^ Intensities were derived via a simple Boltzmann analysis and normalized with respect to the lowest-energy conformer. Interestingly, while the previous studies had some emphasis on the “chair” and “boat” configurations of the proline residue (following the nomenclature from the study on H^+^GPGG),^7^ in the majority of cases only one conformer was found (“boat” in most of these cases), and in cases where both forms were obtained, the lowest Gibbs free energy conformer is reported.

Hydrogen bonds were verified and quantified using the quantum theory of atoms in molecules (QTAIM) as implemented in the Multiwfn computational chemistry package^32^ with the CAM-B3LYP-D3BJ/6-311++G(d,p) densities as validated in the previous study on H^+^XPGG.^8^ The Poincaré–Hopf theorem was found to hold in every case, a necessary condition to ensure no interaction is missed. Hydrogen bonds were verified to exist by three criteria: viz. the bond path, the density at the bond critical point (bcp), and the Laplacian at the bcp. Only bond paths linking a hydrogen to either a nitrogen or an oxygen were recognized as hydrogen bonds, and other interactions were treated as weak interactions beyond the scope of this study. Only bond paths with bcp charge densities above 0.002 a.u. and bcp Laplacians above 0.026 a.u. were considered hydrogen bonds. These values were chosen in accordance with parameters defined by Koch and Popelier for weak hydrogen bonds;^33^ however, they were modified slightly. The original work reported a window of ranges, and a good number of the particularly strong and charge-assisted hydrogen bonds in this report exceed the upper limit of these windows, so the upper limit was removed. Additionally, the cutoff value for the Laplacian was adjusted from 0.024 to 0.026 a.u., and this was done to omit a small number of interactions with extremely unfavorable angles. This change in the Laplacian cutoff slightly affects the *R*^2^ and *r*_s_ values reported in this study (less than 0.015 units); however, it should be reported for consistency. To assess the relative strengths of hydrogen bonds, the potential energy density, *V*(*r*), at the bcp was used as it was in the previous H^+^XPGG study.^8^ We again note that results on experimental electron densities have led to a relationship between hydrogen-bond energies and the value of *V*(*r*);^34^ we report the raw *V*(*r*) value at the bcp and speak in ratios of potential energy densities to assess relative hydrogen-bond strengths. All potential energy densities, charge densities, and Laplacians for all hydrogen bonds in each conformer/tautomer for each of the methylated and unmethylated versions of H^+^KPGG can be found in the Supporting Information.

### 2.2 Experimental Details

KPGG was synthesized through standard Fmoc solid-phase peptide synthesis using Fmoc-protected amino acids and Fmoc-Gly-Wang resin (Midwest Biotech, Fishers, Indiana). Deprotection was performed with 20% piperidine in dimethylformamide, and 1,3-diisopropylcarbodiimide and 6-chloro-1-hydroxybenzotriazole were used as coupling reagents. KPGG was cleaved from the resin using an 18:1:1 ratio of trifluoroacetic acid/triisopropylsilane/methanol. KPGG was precipitated into, and washed using, ice cold ether then dried and used without further purification. Purity was estimated to be >90% by MS analysis.

Electrospray solutions were prepared to ∼10 μM in 50:50 water/methanol. IMS theory^31,35−37^ and instrumentation^38^ are provided in detail elsewhere. Briefly, ions were produced by electrospray ionization (TriVersa NanoMate autosampler, Advion, Ithaca, New York) and then transferred and stored in an ion funnel trap at the entrance to the IMS-MS instrument.^38,39^ The gate is periodically opened for ∼75 μs to release ion packets into the 3 meter drift tube filled with 3.00 ± 0.03 Torr He buffer gas, held at ∼10 V cm^–1^. The ion mobilities are determined by measuring the time required to traverse the drift tube, *t*_D_, and then related to collision cross section, Ω, by eq 1.^31,40−42^
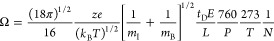
1Furthermore, in eq 1, ze is the charge on the ion, *E* is the electric field, *L* is the length of the drift tube, *P* and *T* are the gas pressure and temperature, and *N* is the neutral number density of the buffer gas (at STP). *m*_I_ and *m*_B_ are the masses of the ion and buffer gas, respectively. Structures with large collision cross sections (CCSs) interact with the buffer gas more often than compact species, resulting in a difference in drift time (*t*_D_) through the IMS cell. Mobility-separated ions exit the drift tube and are pulsed orthogonally into a time-of-flight mass spectrometer for analysis of their mass-to-charge (*m*/*z*) ratios. As in our previous work, the ion storage conditions were adjusted to reflect the gas-phase quasi-equilibrium distributions.^43^

## 3 Results and Discussion

### 3.1 Entropy Chooses the Preferred Tautomer of H^+^KPGG

**Figure 1:**
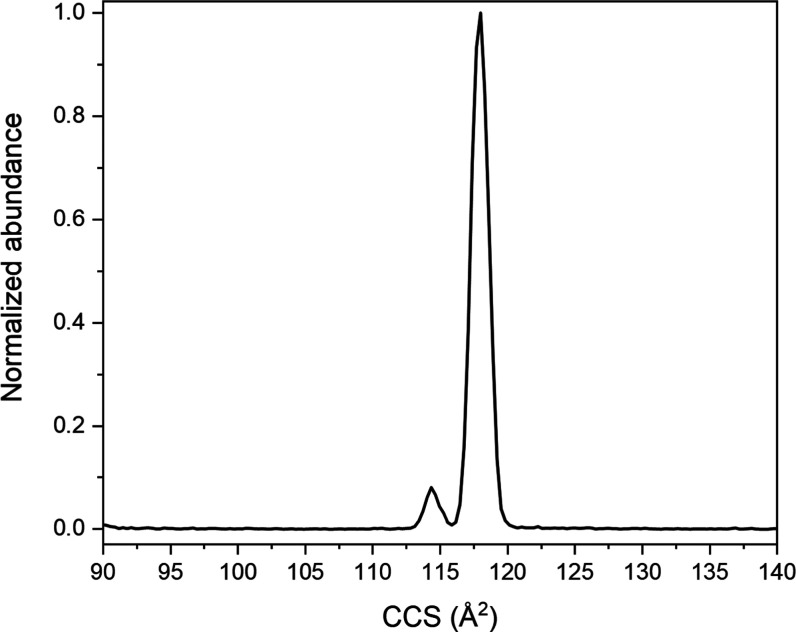
Experimental collision cross-section distribution of singly protonated KPGG.

**Figure 2:**
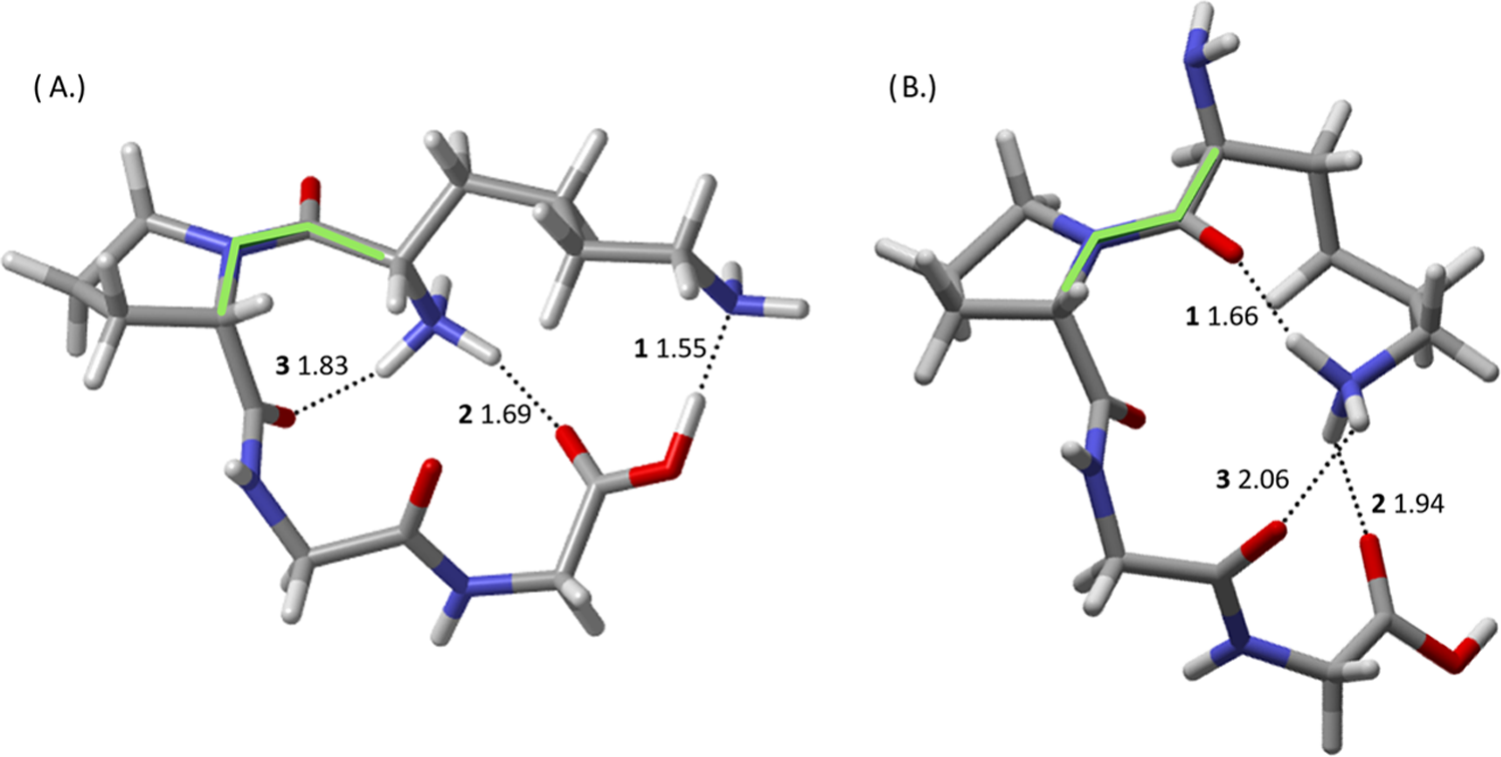
CAM-B3LYP-D3BJ/6-311++G(d,p)-optimized geometries of the two lowest-energy H^+^KPGG tautomers: (A) Nt-*cis*-1, with the additional proton located on the N-terminal amino group, and (B) K-*trans*-1, with the additional proton located on the lysine side-chain amino group. Dotted lines indicate hydrogen bonds determined through QTAIM as described in Section 2, distances in angstroms, and bold numbers referring to hydrogen-bond rank order labeled from strongest to weakest, ascending. Light green line drawn to illustrate *cis* vs *trans* geometries of the proline and N-terminal α carbons.

Figure 1 shows the experimentally obtained ion mobility spectrum of H^+^KPGG. The spectrum is dominated by a single peak at 117.9 Å^2^ and a minor feature at 114.3 Å^2^ with a normalized intensity of 8.1% relative to the larger peak. From the previous two studies on proline-containing hairpins, it is clear that quantum chemical calculations are necessary to elucidate the structures corresponding to each peak. We used quantum chemical calculations, as outlined in Section 2, to determine optimized structures of the two dominant species that are consistent with the CCS values measured in Figure 1; Figure 2A corresponds to the minor peak and Figure 2B corresponding to the major feature. Unlike spectra of singly protonated GPGG, DPGG, NPGG, EPGG, and QPGG, the species making up the experimental IMS spectrum of H^+^KPGG are tautomers differing in the placement of the excess proton.^8^

The conformer making up the minor peak, Figure 2(A), will be referred to throughout this report as Nt-*cis*-1, with Nt referring to the placement of the excess proton on the N-terminal amino group, *cis* referring to the *cis* orientation of the Pro and Lys α carbons (illustrated by the green line in Figure 2(A)), and −1 referring to this being the lowest-lying Nt-*cis* conformer in terms of Gibbs free energy. Of relevance to the previous studies, Nt-*cis*-1 corresponds to the *cis*-1r structures reported in the study of H^+^XPGG, where X = D, N, E, and Q, with −1*r* corresponding to a specific arrangement of the hydrogen-bonding network as well as an interaction between the C-terminal hydroxyl group and the N-terminal residue’s side chain.^8^ However, here we are more interested in the tautomers rather than individual conformers and number the structures of any given conformer of each tautomer by relative Gibbs free energies. The conformer making up the dominant peak, Figure 2(B), will be referred to as K-*trans*-1, with the prefix K referring to tautomers wherein the excess proton is located on the lysine side-chain amino group, *trans* referring to the *trans* orientation of the Pro and Lys α carbons (illustrated by the green line in Figure 2(B)), and −1 referring to the fact that this is the lowest-lying conformer with the proton on the lysine side chain. There is no simple analogue for K-*trans*-1 available in the previous studies as here all hydrogen bonds are formed with the side chain and the typical *trans* structure is disrupted by the Gly-3 carbonyl group engaging in a hydrogen bond with the protonated side-chain amino rather than the C-terminal hydroxyl group (K-*trans*-1 hydrogen bond **3**).^8^

The conformational preference of both tautomers is quite strong: in all of the lowest-lying conformers examined in this report (as well as for the methylated lysine analogues), the side-chain-protonated species prefer the *trans* orientation and the N-terminally protonated species prefer the *cis* orientation. The *cis* favorability of the N-terminally protonated tautomer is expected, especially when considering the overall stability of *cis* conformers vs *trans* conformers when side-chain interactions are present.^8^ The *trans* favorability of the side-chain-protonated tautomer, at least for the preferred conformer, is likely to allow hydrogen bonds with the Lys carbonyl (K-*trans*-1 **1**) in tandem with hydrogen bonds with the carbonyl groups on the opposite side of the proline ring (K-*trans*-1 **2** and **3**). Not all low-lying conformers of the side-chain-protonated tautomer possess the equivalent hydrogen bond of K-*trans*-1 **1** (of the 10 listed in the Supporting Information five do not); however, in those cases, it is likely a simple case of steric effects, with the *cis* configuration orienting the side chain so it can only interact with the C-terminus without significant distortion.

Table 1 details the comparison between theory and experiment for the ion mobility spectrum of H^+^KPGG. The theoretical collision cross sections agree to within <0.2%, of the experimental values, well below the uncertainties expected for calculated CCSs from trajectory methods using MOBCAL.^44^ The agreement between the theoretical and experimental cross sections cements Nt-*cis*-1 as the minor peak and K-*trans*-1 as the major peak, with no other conformers needed to describe the spectrum fully. The final Gibbs free energies align with experiment quite well, with Nt-*cis*-1 predicted to be the minor conformer with a calculated intensity of 29.9% compared to 8.1% experimentally. An 8.1% normalized intensity corresponds to a Gibbs-free-energy difference of 1.49 and 0.77 kcal/mol greater than reported here, producing an error well within chemical accuracy (2 kcal/mol). Additionally, previous work benchmarking gas-phase basicities of amino acids reported the difference between the lowest-energy N-terminally protonated conformer and lowest-energy side-chain-protonated conformer of lysine to be 3.6 kcal/mol (14.9 kJ/mol) at the CBS-QB3 level of theory (available in the Supporting Information of the cited ref 1). This is over twice the value we see here, which can be rationalized by considering the larger wealth of hydrogen-bonding opportunities in a multiresidue peptide equalizing the two tautomers.

**Table 1:** Experimental and Theoretical IMS Collision Cross Sections and Intensities of H^+^KPGG (%)^a^

species	exp. CCS	theory CCS	exp. %	Δ*E*_0_ (%)	Δ*H* (%)	Δ*G* (%)
Nt-*cis*-1	114.3	114.3	8.1%	0 (100%)	0 (100%)	0.72 (29.9%)
K-*trans*-1	117.9	118.1	100%	1.48 (8.3%)	2.13 (2.7%)	0 (100%)

a Theoretical cross sections averaged over 100 random number seeds, relative thermochemical quantities reported in kcal/mol with normalized Boltzmann populations in parentheses, calculated as described in the Methods section. Δ*E*_0_ refers to zero-point corrected energies, Δ*H* refers to enthalpies, and Δ*G* refers to Gibbs free energies. CCS in Å^2^.

In line with the previous H^+^GPGG study,^7^ the difference between the *cis* and *trans* conformers is entropically driven. However, given that this is a tautomerization and that there is a drastic difference in the hydrogen-bonding network, the entropy difference (2.84 kcal/mol) is larger than that in the H^+^GPGG case (1.84 kcal/mol at the CAM-B3LYP/6-311++G(d,p) level of theory used here). While a difference varying by a single kcal/mol is not a large amount energetically, in terms of the calculated entropy this can be a cause for concern. The vibrational entropy is dependent on low frequencies which are also the quantities most likely to be affected by anharmonicity. Previous work outlined how frequency scaling, the most common method for correcting this deficiency, does not significantly affect the overall conformational energies even in drastic circumstances.^7^ However, given the previous work on D, N, E, and QPGG, this is a good opportunity to explore the effects of hydrogen-bonding differences on the entropy.^8^ Finding a correlation between hydrogen-bonding strength and entropy would be interesting not only from a purely theoretical point of view but also in validating and motivating the use of uncorrected entropic differences between skeletally similar conformers here and in the future. Additionally, neither lysine nor the protonated forms of any of the other reported amino acids, calculated with CBS-QB3 in the most recent, cohesive, amino acid benchmarking study, were found to change the preferred tautomer (or conformer, for that matter) when considering the entropic contribution at room temperature.^1^ Here, we see that not just in conformational searches, as seen with H^+^GPGG, but when considering separate tautomers as well, that entropy plays a key role in the gas-phase peptide structure and that these contributions will likely only increase in importance as the system size grows.

### 3.2 Hydrogen-Bonding Strength Correlates with Entropic Differences

To begin analyzing the effect of hydrogen bonding on the entropic differences reported in the previous section, Table 2 details the potential energy density, *V*(*r*), at the bond critical point (bcp) for each hydrogen bond of both tautomers shown in Figure 2, ordered from strongest to weakest. The previous study on D, N, E, and QPGG detailed the correlation between hydrogen-bond length and *V*(*r*), employing this quantity to detail changes in the hydrogen-bonding networks of *cis* species as the N-terminal residue was changed.^8^ Compared to the intricate and bifurcated hydrogen-bonding networks detailed in the previous studies, the hydrogen-bonding patterns seen in Figure 2 are refreshingly simple.

**Table 2:** BCP Potential Energy Densities of H^+^KPGG Hydrogen Bonds^a^

H-bond	K-*trans*-1	Nt-*cis*-1
**1**	–0.0474	–0.0822
**2**	–0.0187	–0.0408
**3**	–0.0146	–0.0272

a Reported in a.u., hydrogen bonds numbered as in Figure 2.

K-*trans*-1 possesses three hydrogen bonds, each with the protonated side-chain ammonium group as the donor and a carbonyl group as the receptor. The strongest K-*trans*-1 hydrogen bond by far, stronger than the other two combined, has the carbonyl group of the N-terminus acting as the receptor. The strength of this hydrogen bond, **1**, arises likely due to simple steric effects and not as much due to the specific carbonyl being a better hydrogen-bond acceptor than the other carbonyl groups. The N-terminal carbonyl is much more stationary than the other two carbonyl groups participating in hydrogen bonds, making it a good candidate for a fully optimized hydrogen bond (the donor–hydrogen–acceptor bond angle being 163°) with the other two hydrogen bonds being optimized only as far as steric effects will allow.

The protonated N-terminus of Nt-*cis*-1 engages in two hydrogen bonds, one with the Gly-3 carbonyl oxygen and the other with the C-terminal carbonyl oxygen. Both of these hydrogen bonds are eclipsed, however, by the hydrogen bond between the C-terminal hydroxyl group and the lysine side-chain amino group. This single hydrogen bond, **1**, is over twice the value of the next strongest quantity and stronger than the two remaining N-terminal hydrogen bonds combined. An interesting feature of this OH···N hydrogen bond, aside from its strength due to the acceptor/donor pair, is the length of the hydroxyl O–H bond. The O–H bond length in Nt-*cis*-1 is 1.05 Å while the same bond in K-*trans*-1, where it is not involved in any hydrogen bonding, is 0.97 Å, an appreciable difference. The strength of this hydrogen bond and the resulting length of the hydroxyl O–H bond mark this as a good candidate interaction for a salt-bridge interaction, as has been seen in previous work on lysine by itself.^1^ However, resonance destabilization by the N-terminal hydrogen bond with the C-terminal carbonyl likely plays a role in making deprotonation of the hydroxyl group energetically unfavorable enough to prevent formation of a full salt-bridge interaction. Interestingly, and in line with this theory, a higher-energy N-terminally protonated conformer (Nt-*cis-*3) sees the lysine amino group form a salt bridge by deprotonation of the C-terminal hydroxyl group and the hydrogen bond between the C-terminus and the protonated N-terminus is weaker in this case (see the Supporting Information). Even without a full salt bridge, however, the total potential energy density from hydrogen bonding in Nt-*cis*-1 is nearly twice the value of K-*trans*-1. This difference can be explained not only by the particularly strong hydrogen bond with the lysine amino group but also by the two remaining hydrogen bonds with the protonated nitrogen being allowed to optimize more fully due to the lack of a third hydrogen bond constraining rotation of the N-terminus, as it does in K-*trans*-1.

**Figure 3:**
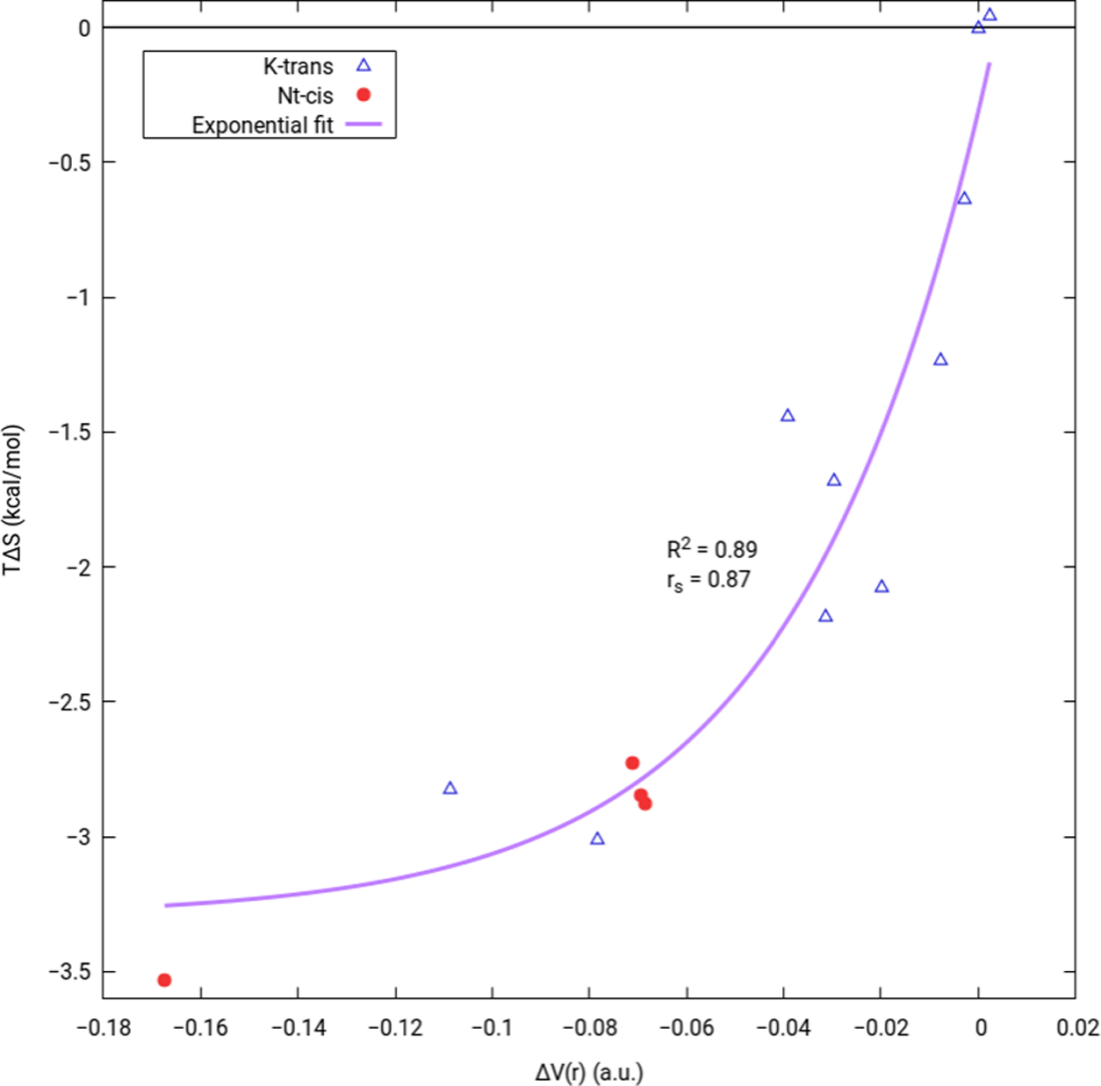
Relative entropic contribution, *T*Δ*S*, *T* = 298.15 K, vs relative potential energy density, Δ*V*(*r*), of all verified hydrogen bonds, summed. Plotted for 14 of the lowest-energy conformers/tautomers of H^+^KPGG relative to K-*trans*-1 with side-chain-protonated species as blue triangles and N-terminally protonated species as red circles. Exponential fit (form: *n *exp(*x*/*u*) + *b*) in purple with displayed *R*^2^ and *r*_s_ (Spearman’s rank-order correlation coefficient). Structures for all conformers and QTAIM data for all hydrogen bonds can be found in the Supporting Information.

An interesting result to be gleaned from Tables 1 and 2 is that the entropically disfavored tautomer, Nt-*cis*-1, exhibits a much stronger hydrogen-bond network than the entropically favored specimen. This result implies that a tighter hydrogen-bond network affects the vibrational entropy through a blue shift of low-frequency modes that rely on collective torsions across the hairpin peptide, resulting in a lower entropy compared to less-constrained conformers. In this way, a small number of hydrogen bonds can drastically affect the entropy difference between two conformers or tautomers. Two datapoints, however, do not make a trend. To further explore this idea, Figure 3 plots the relative (compared to K-*trans*-1) entropy contribution at room temperature, in kcal/mol, against the relative potential energy density, Δ*V*(*r*), of all hydrogen bonds in a given conformer for the 14 lowest-Gibbs-free-energy conformers of H^+^KPGG. The plotted conformers can be seen in the Supporting Information with associated QTAIM data. Conformers with only minor changes compared to other conformers were discarded, e.g., chair vs boat orientation of the proline or small changes in the alkyl chain for side-chain-protonated conformers. The lack of N-terminally protonated conformers within an appreciable energy window of the lowest-energy conformer led to three conformers differing mostly by alkyl chain dihedrals being plotted.

Figure 3 includes an exponential fit with an *R*^2^ value of 0.89, indicating a good correlation between relative entropic contributions and hydrogen-bonding strengths. Spearman’s rank-order correlation coefficient, *r*_s_, has also been calculated and displayed. A rank-order correlation describes how well the relative rank of *x*-axis data is reflected in the *y*-axis, with changes decreasing the value and 1 indicating perfect agreement. An *r*_s_ of 0.87 as we see here indicates that higher-in-magnitude potential energy densities arising from hydrogen bonds tend to produce lower-in-magnitude entropic contributions. Red circles in Figure 3 indicate N-terminally protonated species while blue triangles correspond to the side-chain-protonated tautomer. The three red circles in the middle of the plot reflect N-terminally protonated conformers that differ mainly by changes in the dihedral angles of the lysine alkyl chain; however, no other N-terminally protonated species were within 4.5 kcal/mol in Gibbs free energy, so these were included for completion. We do not expect such slight changes in geometry to be captured by this model, but note the impressive correlation nonetheless.

Figure 3 is similar to the plot in the previous H^+^XPGG study implicating changes in the *cis*/*trans* energy differences across multiple peptides to differences in the hydrogen-bond potential energy densities.^8^ It should be noted, however, that the quantities being plotted in the previous study were cases where only hydrogen-bond lengths were changed upon substitution with a different amino acid, with small exceptions. This was sufficient to explore how changing out similar amino acids affects the overall favorability of the *trans* conformer. However, here we relate many different conformers (across two tautomers) to one conformer of one tautomer. Hydrogen bonds change drastically across the conformers seen in Figure 3 and attempts to plot energy changes are not fruitful (the Supporting Information contains plots of the zero-point corrected and Gibbs free energies, with the highest *R*^2^ being 0.10) because many other factors are allowed to vary other than just the strengths of hydrogen bonds. This can even be seen in the previous study where a small jump can be seen between the D, N pair and E, Q pair due to new (albeit weak) hydrogen bonds forming. Here, there is no such restriction on hydrogen-bond forming, breaking, or shifting, yet the entropy is exponentially related with a high correlation.

The exponential relationship is expected in this case, as well, due to the exponential terms relating frequencies to vibrational entropy. Figure 3 demonstrates a powerful relationship between entropy and hydrogen bonding, one relevant to discussions of enthalpy–entropy compensation. However, there are still unanswered questions in this analysis. One is the lack of a salt-bridge interaction in Nt-*cis*-1. If the C-terminal carbonyl hydrogen bond is preventing a salt bridge from forming between the hydroxyl group and the lysine side-chain amino group, what perturbation is necessary to induce a salt bridge in the lowest-energy, N-terminally protonated tautomer? Additionally, is there a way to make the entropically preferred side-chain-protonated tautomer disfavored compared to the alternative? To answer these questions, we move to the purely computational exploration of methylation of the lysine side chain.

### 3.3 Methylation of the Lysine Side Chain Dramatically Alters Relative Electronic Energies

**Figure 4:**
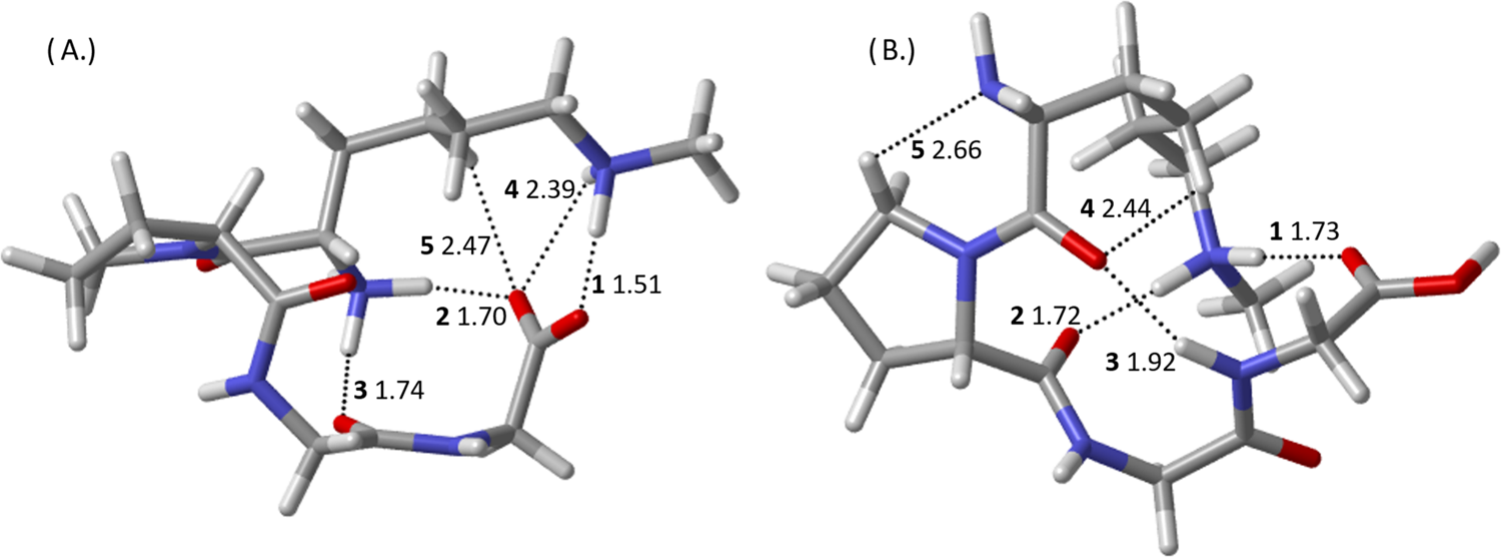
CAM-B3LYP-D3BJ/6-311++G(d,p)-optimized geometries of the lowest-energy (A) N-terminally protonated (Me_1_Nt-*cis*-1) and (B) side-chain-protonated (Me_1_K-*trans*-1), singly methylated H^+^KPGG conformers. Dotted lines indicate hydrogen bonds determined through QTAIM as described in Section 2, distances in Angstroms, and bold numbers referring to hydrogen-bond rank order labeled from strongest to weakest, ascending.

We will begin studying the methylation of the lysine side chain in H^+^KPGG by considering the structures of the individual conformers. Figure 4 shows the lowest-energy side-chain-protonated (Figure 4A, Me_1_Nt-*cis*-1) and N-terminally protonated (Figure 4B, Me_1_K-*trans*-1) conformers of singly methylated H^+^KPGG. QTAIM data on the assigned hydrogen bonds of both tautomers can be found in the Supporting Information, relative strengths will briefly be discussed. Immediately apparent is that the lowest-energy methylated species are more complicated than the lowest-energy unmethylated species with five hydrogen bonds per conformer as opposed to three in the previous section (though higher-energy conformers exhibited a larger number of hydrogen bonds).

The lowest-energy, singly methylated, side-chain-protonated conformer (Figure 4B) exhibits two weak hydrogen bonds from CH donors (Me_1_K-*trans*-1 **4** and **5**), one donating to the N-terminus which went vacant in the unmethylated specimen. The protonated side chain donates to two hydrogen bonds of near-equal strength, one to the C-terminal carbonyl group and the other to the Gly-3 carbonyl group (Me_1_K-*trans*-1 **1** and **2**, respectively). The lysine carbonyl group, which received a hydrogen bond from the protonated side chain in the unmethylated lowest-energy conformer, now receives a hydrogen bond from the Gly-4 amine with about 80% the strength of the corresponding unmethylated interaction (Me_1_K-*trans*-1 **3**, Supporting Information).

The most striking feature of the lowest-energy, singly methylated, N-terminally protonated, conformer (Figure 4A) is the salt-bridge interaction between the lysine side chain and the C-terminus (Me_1_Nt-*cis*-1 **1**). While the interaction between the lysine side-chain amino group was not strong enough to deprotonate the hydroxyl group in the unmethylated analogue, the addition of an electron-donating methyl group increases the polarity of the nitrogen enough to fully transfer the proton (a bond length of 1.09 Å compared to 1.02 Å on the other side-chain N–H bond), in line with previous results on alkali-metal-cationized methyllysine.^15,16^ The resulting salt-bridge interaction is slightly stronger (0.0860 a.u., Supporting Information) than the strongest hydrogen bond seen in the two conformers examined in the previous section (0.0822 a.u., between the hydroxyl group and the lysine side chain). The proton transfer results in a negative charge on the C-terminus, making the C-terminal carbonyl an ideal hydrogen-bond acceptor. Indeed, the second strongest interaction is the charge-assisted hydrogen bond between the protonated N-terminus and the C-terminal carbonyl (Me_1_Nt-*cis*-1 **2**). This is slightly stronger than the unmethylated analogue (by 0.002 a.u.). The C-terminal carbonyl receives two other hydrogen bonds, one from the side-chain amine and one from a C–H donor along the alkyl side chain (Me_1_Nt-*cis*-1 **4** and **5**, respectively). While in this and the previous studies, carbonyl groups mainly receive two hydrogen bonds at most, this saturation is due to the negative charge on the carbonyl and is present in higher-energy conformers plotted in the previous section where the same proton transfer occurs (Supporting Information). Finally, the protonated N-terminus engages in one other hydrogen bond, with the Gly-3 carbonyl (Me_1_Nt-*cis*-1 **3**), slightly weaker than the analogue, hydrogen bond **2**, in Me_1_K-*trans*-1 (0.001 a.u.) due to the effect of the methyl group in the side-chain-protonated conformer but substantially stronger than the unmethylated analogue (a 31% increase, Supporting Information) due to the N-terminus only donating to two hydrogen bonds rather than three.

**Figure 5:**
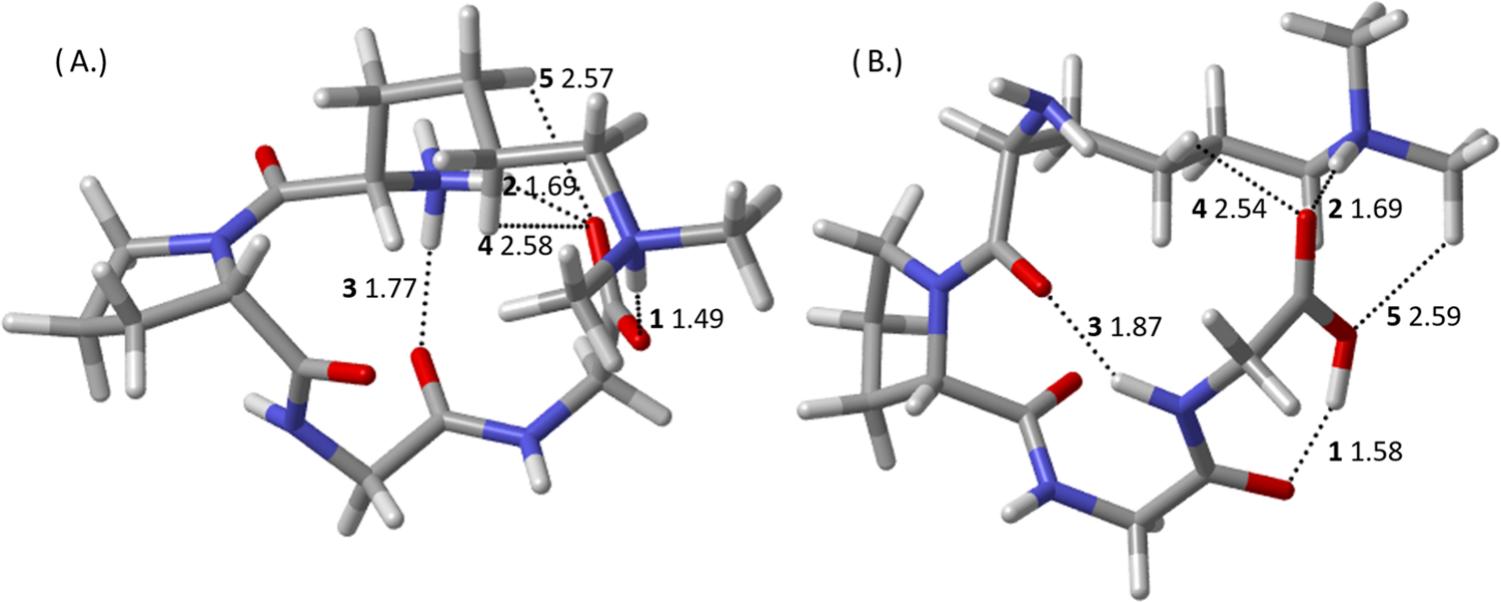
CAM-B3LYP-D3BJ/6-311++G(d,p)-optimized geometries of the lowest-energy (A) N-terminally protonated (Me_2_Nt-*cis*-1) and (B) side-chain-protonated (Me_2_K-*trans*-1), doubly methylated H^+^KPGG conformers. Dotted lines indicate hydrogen bonds determined through QTAIM as described in Section 2, distances in Angstroms, and bold numbers referring to hydrogen-bond rank order labeled from strongest to weakest, ascending.

Addition of a single methyl group has a profound impact on the hydrogen-bond network of H^+^KPGG; however, we expect a second methyl group to exaggerate these effects and report the lowest-energy, doubly methylated, side-chain-protonated (A) and N-terminally protonated (B) conformers of H^+^KPGG in Figure 5. The lowest-energy side-chain-protonated conformer now exhibits only one charge-assisted hydrogen bond donated from the side-chain amine; however, it is shorter and stronger than the two side-chain hydrogen bonds exhibited in the singly methylated analogue (−0.0444 a.u. for Me_2_K-*trans*-1 hydrogen bond **2** compared to −0.0388 and −0.0377 a.u. for **1** and **2** of Me_1_K-*trans*-1, *V*(*r*) data available in the Supporting Information). The C-terminal carbonyl accepts the hydrogen bond from the side-chain amine (Me_2_K-*trans*-1 **2**); however, the position of the carbonyl is able to be optimized and allows the C-terminal hydroxyl group to donate a hydrogen to the Gly-3 carbonyl (Me_2_K-*trans*-1 **1**), a donor and acceptor pair that go without hydrogen bonds in the previous case. Aside from these two examples, the other hydrogen bonds in Me_2_K-*trans*-1 involve weak CH donors and the other moderate hydrogen bond is the C-terminal amine donating to the lysine carbonyl group (Me_2_K-*trans*-1 **3**) in the same fashion as in the singly methylated case (Me_1_K-*trans*-1 **3**).

The lowest-energy, doubly methylated, N-terminally protonated conformer (Me_2_Nt-*cis*-1) is only slightly perturbed from the singly methylated case (Me_1_Nt-*cis*-1). The backbone RMSD between Me_2_Nt-*cis*-1 and Me_1_Nt-*cis*-1 is 0.04 Å, much lower than the 0.65 Å RMSD for Me_1_Nt-*cis*-1 compared to the unmethylated Nt-*cis*-1. The only change in the identity of hydrogen-bond pairs is that the lysine side-chain amine only participates in the salt-bridge interaction (Me_2_Nt-*cis*-1 **1**) and a second CH···O interaction forms between the C-terminal carbonyl and the lysine alkyl chain instead (Me_2_Nt-*cis*-1 **4**). The salt-bridge interaction is strengthened very slightly (0.002 a.u., 0.002 Å shorter), but not enough to fully offset the loss of a charge-assisted hydrogen bond (Table 3).

**Table 3:** Calculated Thermochemical Quantities of Methylated H^+^KPGG Tautomers and Total Hydrogen-Bond Potential Energy Densities^a^

K Me	species	theory CCS	Δ*E*_0_ (%)	Δ*H* (%)	Δ*G* (%)	*V*(*r*)
0	K-*trans*-1	118.1	1.48 (8.3%)	2.13 (2.7%)	0 (100%)	–0.0807
0	Nt-*cis*-1	114.3	0 (100%)	0 (100%)	0.72 (29.9%)	–0.1502
1	K-*trans*-1	118.3	0 (100%)	0 (100%)	0 (100%)	–0.1095
1	Nt-*cis*-1	116.6	2.54 (1.4%)	2.07 (3.1%)	3.19 (0.5%)	–0.1812
2	K-*trans*-1	121.2	2.20 (2.5%)	2.38 (1.8%)	1.96 (3.7%)	–0.1425
2	Nt-*cis*-1	119.4	0 (100%)	0 (100%)	0 (100%)	–0.1780

a Leftmost column enumerates methyl groups attached to the lysine side-chain amino group. Theoretical cross sections averaged over 100 random number seeds, thermochemical quantities in kcal/mol and relative to lowest, with normalized Boltzmann populations in parentheses, calculated as described in Section 2. Total potential energy density summed over all hydrogen bonds, in a.u.; QTAIM data for all hydrogen bonds can be found in the Supporting Information CCS in Å^2^.

Table 3 details the relative thermochemical quantities of the lowest-energy conformers of both tautomers of H^+^KPGG and its singly and doubly methylated analogues. The final column in Table 3 details the sum-total potential energy density arising from the bond critical points of every hydrogen bond in the given conformer. Clearly, the overall strength of the lowest-energy, side-chain-protonated conformer’s (K-*trans*-1) hydrogen-bond network steadily increases with the number of methyl groups added to the side-chain amine. The first increase in overall hydrogen-bond strength arises from the increased number of hydrogen bonds available as well as the loss of a charge-assisted hydrogen bond (Me_1_K-*trans*-1 **2**) being made up by the interaction between the lysine carbonyl and C-terminal amine (Me_2_K-*trans*-1 **3**). The second increase in total hydrogen-bond energy for the side-chain-protonated tautomer (from one methyl group to two) arises from the strong hydrogen bond between the hydroxyl group and Gly-3 carbonyl (Me_2_K-*trans*-1 **1**). Conversely, the total hydrogen-bond strength of the N-terminally protonated tautomer (Nt-*cis*-1) increases with the addition of hydrogen bonds when adding a methyl group; however, a second methyl group slightly lowers the total hydrogen-bond energy through loss of a hydrogen bond that does not afford further flexibility to form new interactions (Me_1_Nt-*cis*-1 **4**). This closes the gap between the side-chain-protonated and N-terminally protonated species in terms of hydrogen-bond strength when the lysine side-chain amine contains two methyl groups, in fact the doubly methylated difference in *V*(*r*) is 51% the original H^+^KPGG value.

When considering one additional methyl group, the side-chain-protonated tautomer is preferred in Gibbs free energy by 3.19 kcal/mol, an over fourfold increase. Additionally, the preference of protonation on the side chain is no longer determined by entropy, with the entropic difference now smaller, slightly over a kcal/mol, making side-chain-protonation entropy independent. It should be noted here that the change in the entropy difference between the two tautomers in the singly methylated case compared to unmethylated do not line up with the change in Δ*V*(*r*), which is slight (0.002 a.u.), as we see in Figure 3. This indicates that the correlation between entropy differences and hydrogen-bond strengths only strongly applies to conformers and tautomers of a single specimen, not across chemical modifications that add additional degrees of freedom affecting low-frequency modes and the vibrational entropy. General trends, such as a much smaller Δ*V*(*r*) resulting in a smaller entropy difference likely still hold; however, strict adherence to the trend demonstrated in Figure 3 is not expected.

Addition of a second methyl group, however, causes the N-terminally protonated tautomer to be favorable in Gibbs free energy by nearly 2 kcal/mol. The entropy difference is quite small between the two tautomers, as is the difference between the zero-point corrected energy and the enthalpy (less than 0.2 kcal/mol compared to nearly 0.5 kcal/mol when singly methylated). This, along with the substantially smaller value of Δ*V*(*r*), indicates that similar hydrogen-bonding strengths cause these tautomers to chiefly differ in terms of electronic energy. As Me_2_Nt-*cis*-1 does not differ greatly from Me_1_Nt-*cis*-1, the change in preference likely corresponds to steric changes in Me_2_K-*trans*-1 compared to its singly methylated analogue.

All three peptides are presented in Table 3 and are useful for separate goals in terms of experimental studies. The tautomerization of H^+^KPGG being entropy driven has the consequence of tautomer population being primarily affected by the temperature with a steep gradient. This can make the relative populations of both tautomers useful for obtaining an experimentally confirmed value for the entropic contribution by slightly adjusting the temperature in an IMS-MS experiment and exploring cases of kinetic trapping. While the overall computational Gibbs-free-energy values correspond well with experiment, obtaining an experimental value for the entropy difference between the tautomers would be invaluable in understanding the thermodynamics of these processes. In terms of methylation, adding a single methyl group to the lysine side-chain amine ensures protonation of the side chain while two methyl groups ensure protonation of the N-terminus. N-terminal protonation with methyl groups on the side chain, however, comes with the caveat of a salt-bridge interaction placing the C-terminal hydrogen on the lysine side chain. To further study these species, and perhaps lead to a system where the proton prefers the N-terminus without a salt-bridge interaction, it would be fruitful to investigate addition of methyl groups to the N-terminus.

## 4 Conclusions

This study began by focusing on the ion mobility distribution of H^+^KPGG. The two IMS peaks were found to correspond not only to one *cis* and one *trans* conformer about the Lys-Pro peptide bond but also to two separate tautomers differing by location of the excess proton. The major peak corresponds to a *trans* orientation and the proton located on the side chain while the minor peak corresponds to a *cis* orientation and a protonated N-terminus. The intensities of the peaks were found through benchmarked DFT calculations to be entropy dependent, with the side-chain-protonated tautomer preferred by in Gibbs free energy but enthalpically disfavored. By including other low-lying conformers/tautomers, it was found that the entropic changes correlate well with changes in the overall hydrogen-bonding strength of a conformer, with stronger hydrogen bonds blue-shifting low-frequency modes and decreasing the vibrational entropy. In order to investigate possible mechanisms for controlling the location of the excess proton, singly and doubly methylated variants of the lysine side chain were studied computationally. Adding a single methyl group was found to make side-chain protonation enthalpically favorable while addition of two methyl groups favored protonation at the N-terminus. These discoveries create a gas-phase toolkit for understanding protonation in peptides and the impacts on the structure in vacuo. This case study for KPGG provides an impetus for furthering developments in temperature-controlled IMS instruments, with the goal of controlling the entropic components that stabilize structure.

## Supporting Information Available

Relative properties of all discussed and plotted conformers and tautomers; further plots relating energetic quantities to hydrogen-bond strengths; labeled images of each H^+^KPGG conformer/tautomer; and full QTAIM information for each bcp in each conformer/tautomer of H^+^KPGG and its methylated analogues (PDF)

## References

[ref1] Uddin K. M.; Warburton P. L.; Poirier R. A. Comparisons of Computational and Experimental Thermochemical Properties of α-Amino Acids. J. Phys. Chem. B 2012, 116, 3220–3234. 10.1021/jp210948m. 22329643

[ref2] Sokalingam S.; Raghunathan G.; Soundrarajan N.; Lee S.-G. A Study on the Effect of Surface Lysine to Arginine Mutagenesis on Protein Stability and Structure Using Green Fluorescent Protein. PLoS One 2012, 7, e40410 10.1371/journal.pone.0040410. 22792305 PMC3392243

[ref3] Shimomura A.; Matsui I.; Hamano T.; Ishimoto T.; Katou Y.; Takehana K.; Inoue K.; Kusunoki Y.; Mori D.; Nakano C.; Obi Y.; Fujii N.; Takabatake Y.; Nakano T.; Tsubakihara Y.; Isaka Y.; Rakugi H. Dietary L-Lysine Prevents Arterial Calcification in Adenine-Induced Uremic Rats. J. Am. Soc. Nephrol. 2014, 25, 1954 10.1681/ASN.2013090967. 24652795 PMC4147981

[ref4] Vaz F. M.; Wanders R. J. A. Carnitine biosynthesis in mammals. Biochem. J. 2002, 361, 417–429. 10.1042/bj3610417. 11802770 PMC1222323

[ref5] Isom D. G.; Castañeda C. A.; Cannon B. R.; García-Moreno E B. Large shifts in pKa values of lysine residues buried inside a protein. Proc. Natl. Acad. Sci. U.S.A. 2011, 108, 5260 10.1073/pnas.1010750108. 21389271 PMC3069169

[ref6] McDonald I. K.; Thornton J. M. Satisfying Hydrogen Bonding Potential in Proteins. J. Mol. Biol. 1994, 238, 777–793. 10.1006/jmbi.1994.1334. 8182748

[ref7] Beckett D.; El-Baba T. J.; Clemmer D. E.; Raghavachari K. Electronic Energies Are Not Enough: An Ion Mobility-Aided, Quantum Chemical Benchmark Analysis of H+GPGG Conformers. J. Chem. Theory Comput. 2018, 14, 5406–5418. 10.1021/acs.jctc.8b00648. 30192543

[ref8] Beckett D.; El-Baba T. J.; Gilbert K.; Clemmer D. E.; Raghavachari K. Untangling Hydrogen Bond Networks with Ion Mobility Spectrometry and Quantum Chemical Calculations: A Case Study on H+XPGG. J. Phys. Chem. B 2019, 123, 5730–5741. 10.1021/acs.jpcb.9b03803. 31241336 PMC6935874

[ref9] Bliznyuk A. A.; Schaefer H. F.; Amster I. J. Proton affinities of lysine and histidine: a theoretical consideration of the discrepancy between experimental results from the kinetic and bracketing methods. J. Am. Chem. Soc. 1993, 115, 5149–5154. 10.1021/ja00065a029.

[ref10] Gronert S.; Simpson D. C.; Conner K. M. A Reevaluation of Computed Proton Affinities for the Common α-Amino Acids. J. Am. Soc. Mass. Spectrom. 2009, 20, 2116–2123. 10.1016/j.jasms.2009.07.006. 19683940

[ref11] Moser A.; Range K.; York D. M. Accurate proton affinity and gas-phase basicity values for molecules important in biocatalysis. J. Phys. Chem. B 2010, 114, 13911–13921. 10.1021/jp107450n. 20942500 PMC2970571

[ref12] Horne D. W.; Broquist H. P. Role of Lysine and ε-N-Trimethyllysine in Carnitine Biosynthesis: I. STUDIES IN NEUROSPORA CRASSA. J. Biol. Chem. 1973, 248, 2170–2175. 10.1016/S0021-9258(19)44201-4. 4266139

[ref13] Vaz F. M.; Wanders R. J. A. Carnitine biosynthesis in mammals. Biochem. J 2002, 361, 417 10.1042/bj3610417. 11802770 PMC1222323

[ref14] Ruthenburg A. J.; Allis C. D.; Wysocka J. Methylation of Lysine 4 on Histone H3: Intricacy of Writing and Reading a Single Epigenetic Mark. Mol. Cell 2007, 25, 15–30. 10.1016/j.molcel.2006.12.014. 17218268

[ref15] Bush M. F.; Forbes M. W.; Jockusch R. A.; Oomens J.; Polfer N. C.; Saykally R. J.; Williams E. R. Infrared Spectroscopy of Cationized Lysine and ε-N-methyllysine in the Gas Phase: Effects of Alkali-Metal Ion Size and Proton Affinity on Zwitterion Stability. J. Phys. Chem. A 2007, 111, 7753–7760. 10.1021/jp071902q. 17636967

[ref16] Bush M. F.; Oomens J.; Williams E. R. Proton Affinity and Zwitterion Stability: New Results from Infrared Spectroscopy and Theory of Cationized Lysine and Analogues in the Gas Phase. J. Phys. Chem. A 2009, 113, 431–438. 10.1021/jp807470p. 19128186

[ref17] Gilbert K. PCMODEL; Serena Software: Bloomington, IN, 2014.

[ref18] Halgren T. A. Merck molecular force field. I. Basis, form, scope, parameterization, and performance of MMFF94. J. Comput. Chem. 1996, 17, 490–519. 10.1002/(SICI)1096-987X(199604)17:5/6<490::AID-JCC1>3.0.CO;2-P.

[ref19] Stewart J. J. P. Optimization of parameters for semiempirical methods V: Modification of NDDO approximations and application to 70 elements. J. Mol. Model. 2007, 13, 1173–1213. 10.1007/s00894-007-0233-4. 17828561 PMC2039871

[ref20] Frisch M. J.; Trucks G. W.; Schlegel H. B.; Scuseria G. E.; Robb M. A.; Cheeseman J. R.; Scalmani G.; Barone V.; Petersson G. A.; Nakatsuji H.; Li X.; Caricato M.; Marenich A. V.; Bloino J.; Janesko B. G.; Gomperts R.; Mennucci B.; Hratchian H. P.; Ortiz J. V.; Izmaylov A. F.; Sonnenberg J. L.; Williams; Ding F.; Lipparini F.; Egidi F.; Goings J.; Peng B.; Petrone A.; Henderson T.; Ranasinghe D.; Zakrzewski V. G.; Gao J.; Rega N.; Zheng G.; Liang W.; Hada M.; Ehara M.; Toyota K.; Fukuda R.; Hasegawa J.; Ishida M.; Nakajima T.; Honda Y.; Kitao O.; Nakai H.; Vreven T.; Throssell K.; Montgomery J. A. Jr.; Peralta J. E.; Ogliaro F.; Bearpark M. J.; Heyd J. J.; Brothers E. N.; Kudin K. N.; Staroverov V. N.; Keith T. A.; Kobayashi R.; Normand J.; Raghavachari K.; Rendell A. P.; Burant J. C.; Iyengar S. S.; Tomasi J.; Cossi M.; Millam J. M.; Klene M.; Adamo C.; Cammi R.; Ochterski J. W.; Martin R. L.; Morokuma K.; Farkas O.; Foresman J. B.; Fox D. J. Gaussian 16, Rev. A.01; Wallingford, CT, 2016.

[ref21] Vosko S. H.; Wilk L.; Nusair M. Accurate spin-dependent electron liquid correlation energies for local spin density calculations: a critical analysis. Can. J. Phys. 1980, 58, 1200–1211. 10.1139/p80-159.

[ref22] Lee C.; Yang W.; Parr R. G. Development of the Colle-Salvetti correlation-energy formula into a functional of the electron density. Phys. Rev. B 1988, 37, 785–789. 10.1103/PhysRevB.37.785. 9944570

[ref23] Becke A. D. Density-functional thermochemistry. III. The role of exact exchange. J. Chem. Phys. 1993, 98, 5648–5652. 10.1063/1.464913.

[ref24] Stephens P. J.; Devlin F. J.; Chabalowski C. F.; Frisch M. J. Ab Initio Calculation of Vibrational Absorption and Circular Dichroism Spectra Using Density Functional Force Fields. J. Phys. Chem. A 1994, 98, 11623–11627. 10.1021/j100096a001.

[ref25] Yanai T.; Tew D. P.; Handy N. C. A new hybrid exchange–correlation functional using the Coulomb-attenuating method (CAM-B3LYP). Chem. Phys. Lett. 2004, 393, 51–57. 10.1016/j.cplett.2004.06.011.

[ref26] Grimme S.; Antony J.; Ehrlich S.; Krieg H. A consistent and accurate ab initio parametrization of density functional dispersion correction (DFT-D) for the 94 elements H-Pu. J. Chem. Phys. 2010, 132, 154104 10.1063/1.3382344. 20423165

[ref27] Goerigk L.; Grimme S. A thorough benchmark of density functional methods for general main group thermochemistry, kinetics, and noncovalent interactions. Phys. Chem. Chem. Phys. 2011, 13, 6670–6688. 10.1039/c0cp02984j. 21384027

[ref28] Grimme S.; Ehrlich S.; Goerigk L. Effect of the damping function in dispersion corrected density functional theory. J. Comput. Chem. 2011, 32, 1456–1465. 10.1002/jcc.21759. 21370243

[ref29] Cato M. A. Jr; Majumdar D.; Roszak S.; Leszczynski J. Exploring Relative Thermodynamic Stabilities of Formic Acid and Formamide Dimers – Role of Low-Frequency Hydrogen-Bond Vibrations. J. Chem. Theory Comput. 2013, 9, 1016–1026. 10.1021/ct300889b. 26588744

[ref30] Copeland C.; Menon O.; Majumdar D.; Roszak S.; Leszczynski J. Understanding the influence of low-frequency vibrations on the hydrogen bonds of acetic acid and acetamide dimers. Phys. Chem. Chem. Phys. 2017, 19, 24866–24878. 10.1039/C7CP04224H. 28869271

[ref31] Mesleh M. F.; Hunter J. M.; Shvartsburg A. A.; Schatz G. C.; Jarrold M. F. Structural Information from Ion Mobility Measurements: Effects of the Long-Range Potential. J. Phys. Chem. A 1996, 100, 16082–16086. 10.1021/jp961623v.

[ref32] Lu T.; Chen F. Multiwfn: A multifunctional wavefunction analyzer. J. Comput. Chem. 2012, 33, 580–592. 10.1002/jcc.22885. 22162017

[ref33] Koch U.; Popelier P. L. A. Characterization of C-H-O Hydrogen Bonds on the Basis of the Charge Density. J. Phys. Chem. A 1995, 99, 9747–9754. 10.1021/j100024a016.

[ref34] Espinosa E.; Molins E.; Lecomte C. Hydrogen bond strengths revealed by topological analyses of experimentally observed electron densities. Chem. Phys. Lett. 1998, 285, 170–173. 10.1016/S0009-2614(98)00036-0.

[ref35] Mason E. A.; M M E. W. Transport Properties of Ions in Gases; John Wiley & Sons, Inc.: New York, New York, 1988; p 90.

[ref36] Mack E. Average cross-sectional areas of molecules by gaseous diffusion methods. J. Am. Chem. Soc. 1925, 47, 2468–2482. 10.1021/ja01687a007.

[ref37] Shvartsburg A. A.; Jarrold M. F. An exact hard-spheres scattering model for the mobilities of polyatomic ions. Chem. Phys. Lett. 1996, 261, 86–91. 10.1016/0009-2614(96)00941-4.

[ref38] Merenbloom S. I.; Koeniger S. L.; Valentine S. J.; Plasencia M. D.; Clemmer D. E. IMS–IMS and IMS–IMS–IMS/MS for Separating Peptide and Protein Fragment Ions. Anal. Chem. 2006, 78, 2802–2809. 10.1021/ac052208e. 16615796

[ref39] Tang K.; Shvartsburg A. A.; Lee H.-N.; Prior D. C.; Buschbach M. A.; Li F.; Tolmachev A. V.; Anderson G. A.; Smith R. D. High-Sensitivity Ion Mobility Spectrometry/Mass Spectrometry Using Electrodynamic Ion Funnel Interfaces. Anal. Chem. 2005, 77, 3330–3339. 10.1021/ac048315a. 15889926 PMC1829302

[ref40] Wyttenbach T.; Helden Gv.; Batka J. J.; Carlat D.; Bowers M. T. Effect of the long-range potential on ion mobility measurements. J. Am. Soc. Mass. Spectrom. 1997, 8, 275–282. 10.1016/S1044-0305(96)00236-X.

[ref41] Jarrold M. F.; Constant V. A. Silicon cluster ions: Evidence for a structural transition. Phys. Rev. Lett. 1991, 67, 2994–2997. 10.1103/PhysRevLett.67.2994. 10044611

[ref42] Clemmer D. E.; Jarrold M. F. Ion Mobility Measurements and their Applications to Clusters and Biomolecules. J. Mass Spectrom. 1997, 32, 577–592. 10.1002/(SICI)1096-9888(199706)32:6<577::AID-JMS530>3.0.CO;2-4.

[ref43] Pierson N. A.; Valentine S. J.; Clemmer D. E. Evidence for a quasi-equilibrium distribution of states for bradykinin [M + 3H]3+ ions in the gas phase. J. Phys. Chem. B 2010, 114, 7777–7783. 10.1021/jp102478k. 20469905 PMC2922466

[ref44] Siu C.-K.; Guo Y.; Saminathan I. S.; Hopkinson A. C.; Siu K. W. M. Optimization of Parameters Used in Algorithms of Ion-Mobility Calculation for Conformational Analyses. J. Phys. Chem. B 2010, 114, 1204–1212. 10.1021/jp910858z. 20039660

