# Supplementary material for: Tautomerization of H+KPGG: Entropic Consequences of Strong Hydrogen-Bond Networks in Peptides
Source: J Phys Chem A. 2023 Jul 25;127(30):6282–91. doi: 10.1021/acs.jpca.3c03744 (PMC10405267; doi:10.1021/acs.jpca.3c03744)
Supplement: Supplementary file 1 — jp3c03744_si_001.pdf [file jp3c03744_si_001.pdf]

Tautomerization of H^+KPGG: Entropic Consequences of Strong Hydrogen Bond Networks in Peptides

Daniel Beckett,[†,‡,*] Tarick J. El-Baba,[‡] Zhichao Zhang,[‡] David E. Clemmer,[‡] and Krishnan Raghavachari[‡]

[†]*Department of Chemistry, Chicago Center for Theoretical Chemistry, The University of Chicago, Chicago, IL 60637*

[‡]*Department of Chemistry, Indiana University, Bloomington, Indiana, 47405*

Supporting Information

Table of Contents

1. Relative Properties of H^+KPGG Conformers……………………………………………….………S2

2. Further Plots With Hydrogen Bonding Strength…………………………………..………..S3

3. All Discussed H+KPGG Conformers……………………………………………………….S5

4. QTAIM Data for H+KPGG and Methylated Analogues……………………………………….S8

S1. H^+KPGG Conformer Relative Energies

Table S1. Relative Properties of H^+KPGG Conformers[a]

Species	Theory CCS	ΔE_0	ΔH	ΔG	$T\Delta S$	$\Delta V(r)$
K-trans-1	117.9	0	0	0	0	0
Nt-cis-1	114.3	−1.476	−2.134	0.715	−2.849	−0.0695
K-trans-2	117.4	0.030	−0.086	1.143	−1.229	−0.0076
Nt-cis-2	115.8	−1.108	−1.713	1.162	−2.875	−0.0686
K-trans-3	114.0	−0.158	−0.430	1.249	−1.679	−0.0296
K-trans-4	118.7	1.361	1.393	1.343	0.050	0.0024
K-trans-5	113.0	−0.388	−0.734	1.448	−2.182	−0.0314
K-trans-6	118.1	0.511	0.205	1.643	−1.438	−0.0393
K-trans-7	112.1	0.112	−0.318	1.752	−2.070	−0.0198
K-trans-8	114.0	−0.036	−0.876	1.943	−2.819	−0.1086
Nt-cis-3	111.2	−0.561	−1.371	2.159	−3.530	−0.2481
K-trans-9	115.4	1.636	1.567	2.201	−0.634	−0.0028
Nt-cis-4	115.7	0.305	−0.267	2.460	−2.727	−0.0710
K-trans-10	113.6	0.344	−0.439	2.567	−3.006	−0.0783

[a] Collision cross sections in square Angstroms, thermochemical properties relative to K-*trans*-1, kcal/mol, temperature 298.15 K, V(r) summed over all hydrogen bonds and relative to K-*trans*-1 in a.u.

S2. Further Plots with Hydrogen Bonding Strength

These plots are included to highlight the lack of correlation between relative energies and relative hydrogen bonding strengths. Linear fit correlates better than exponential fit in both cases.

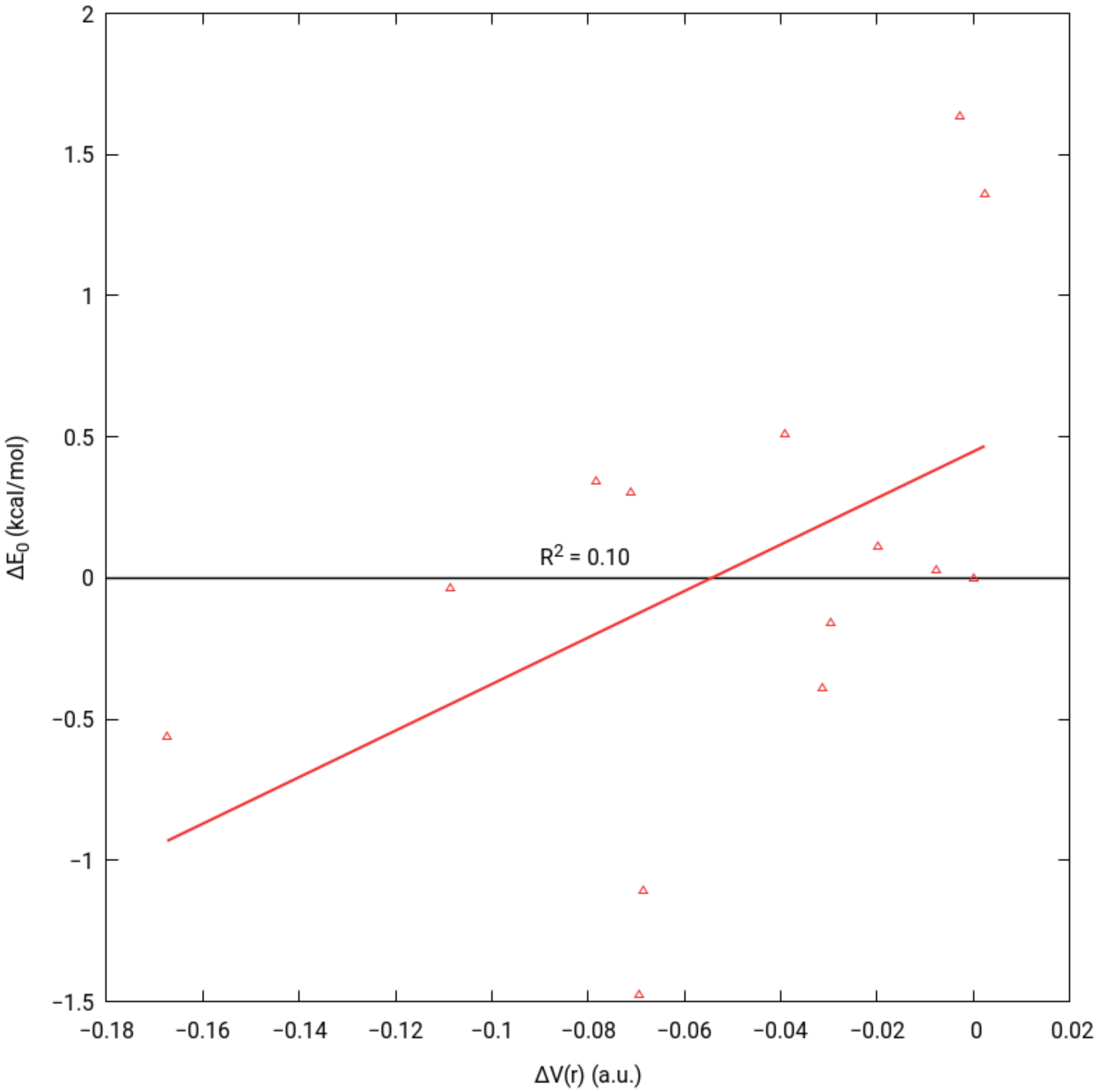

Figure S1. Relative zero-point corrected energy, ΔE_0, vs relative potential energy density, ΔV(r), of all verified hydrogen bonds, summed. Plotted for 14 of the lowest-energy conformers/tautomers of H^+KPGG. Linear fit in red with displayed R^2.

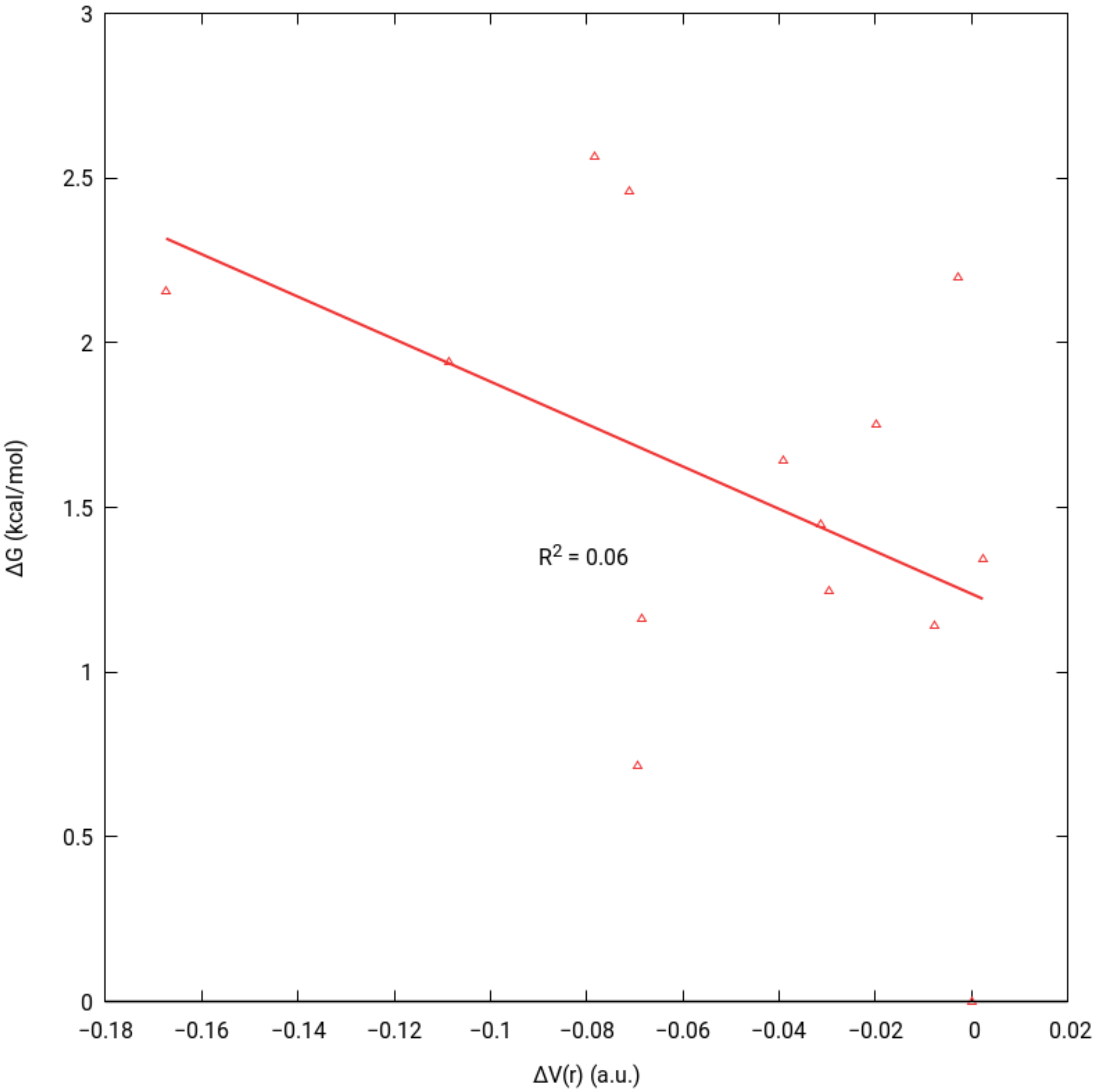

Figure S2. Relative Gibbs free energy, ΔG, vs relative potential energy density, ΔV(r), of all verified hydrogen bonds, summed. Plotted for 14 of the lowest-energy conformers/tautomers of H^+KPGG. Linear fit in red with displayed R^2.

S3. All Discussed H^+KPGG Conformers

Distances displayed in Angstroms for each hydrogen bond, with the bold number denoting the associated identification number of each bond, ordered by strength.

Nt-cis-1

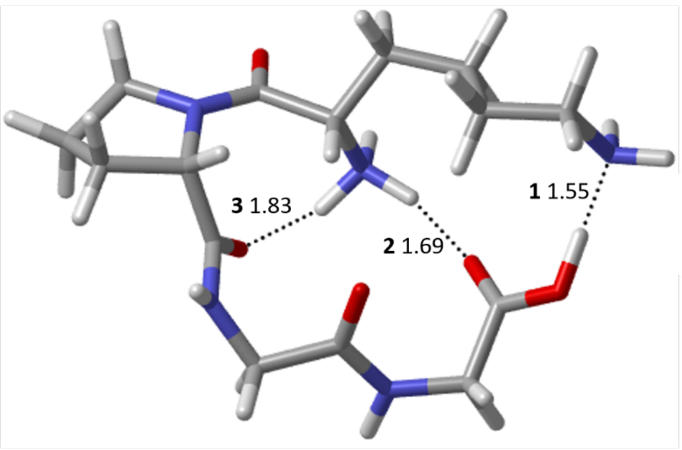

K-trans-1

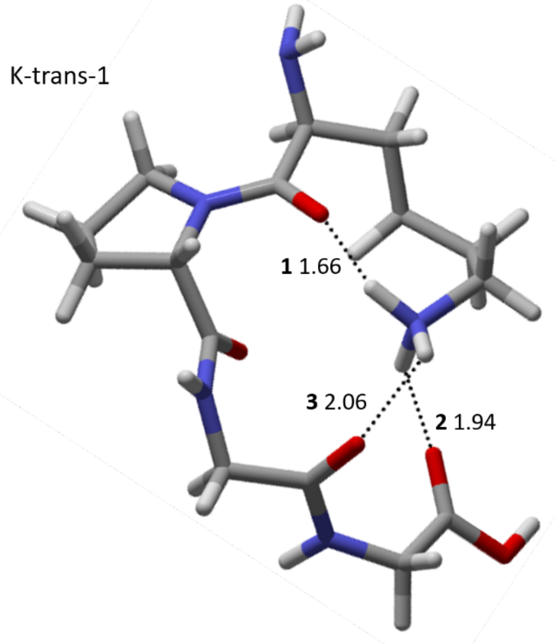

K-trans-2

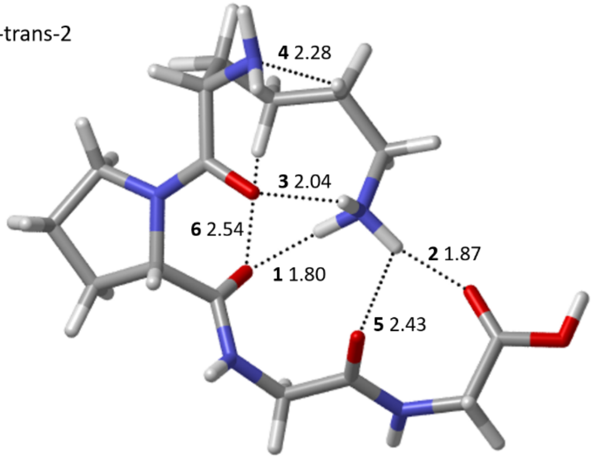

Nt-cis-2

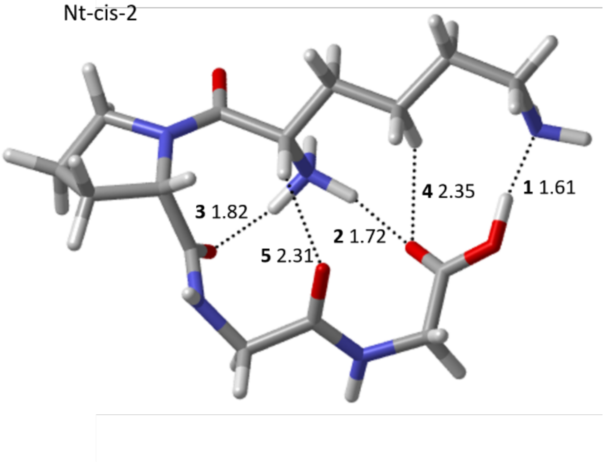

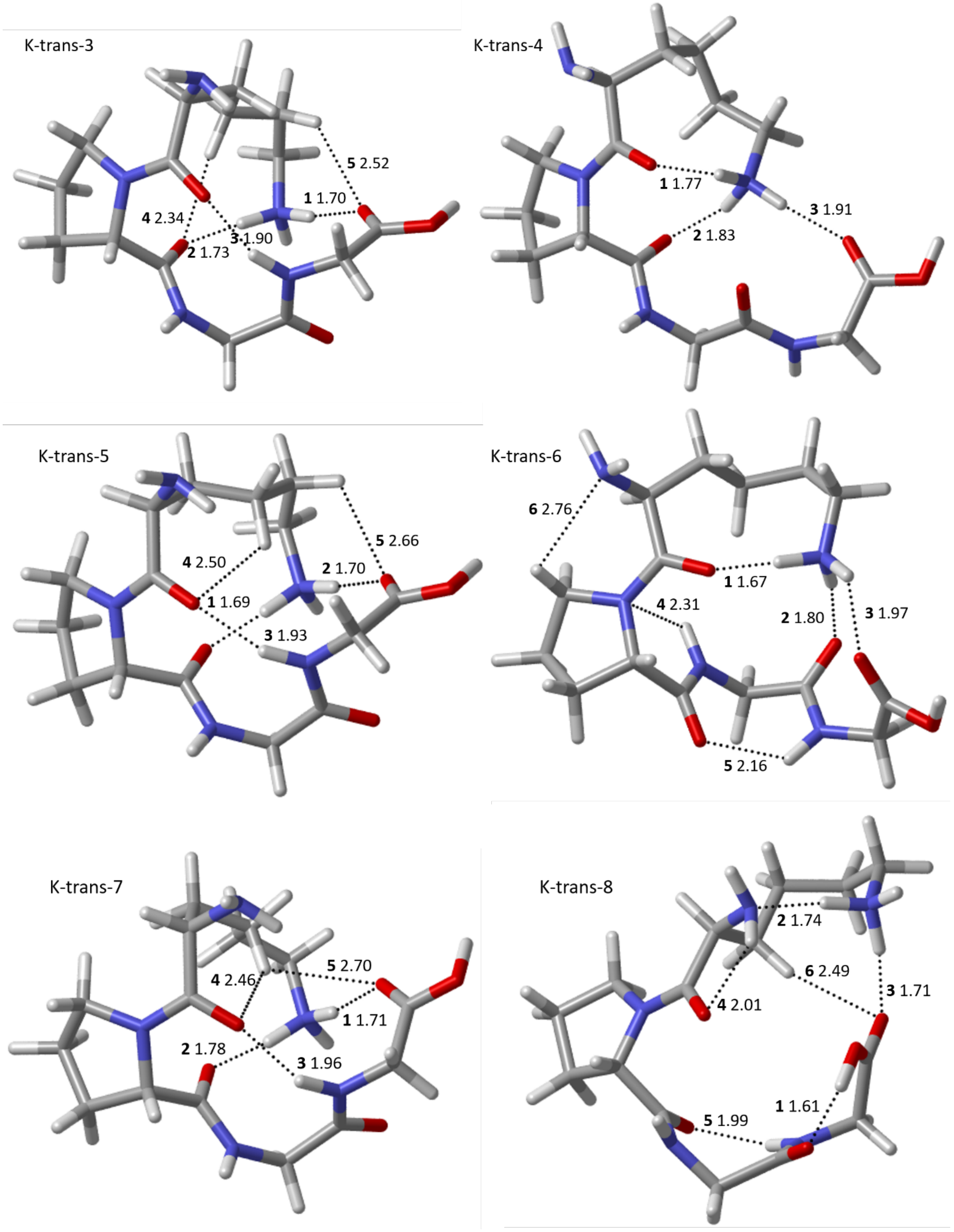
K-trans-3
5 2.52
1 1.70
4 2.34
3 1.90
2 1.73
K-trans-4
1 1.77
3 1.91
2 1.83
K-trans-5
5 2.66
4 2.50
2 1.70
1 1.69
3 1.93
K-trans-6
6 2.76
1 1.67
4 2.31
2 1.80
3 1.97
5 2.16
K-trans-7
5 2.70
4 2.46
1 1.71
2 1.78
3 1.96
K-trans-8
2 1.74
6 2.49
3 1.71
4 2.01
1 1.61
5 1.99

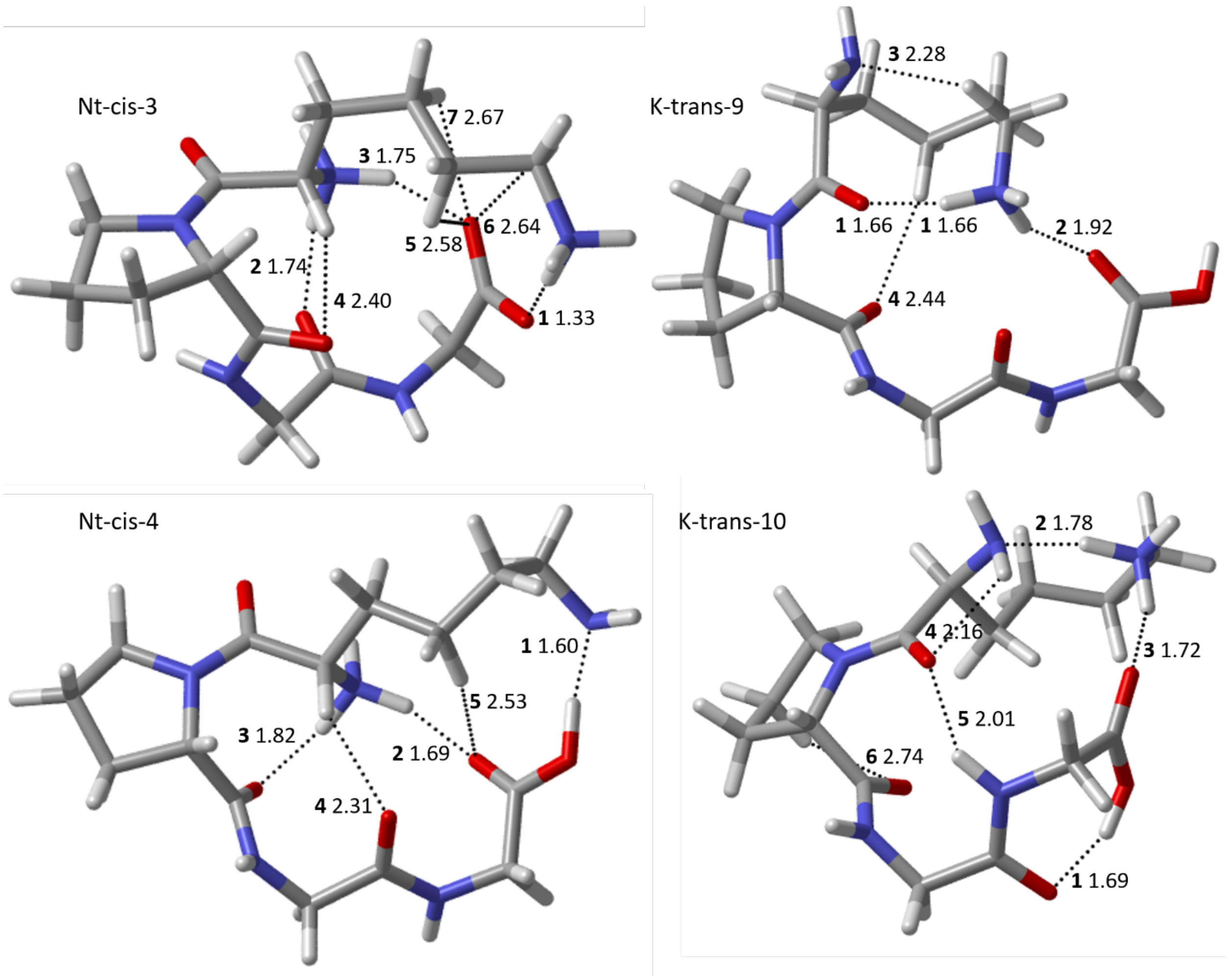
Nt-cis-3
7 2.67
3 1.75
5 2.58
6 2.64
2 1.74
4 2.40
1 1.33
K-trans-9
3 2.28
1 1.66
1 1.66
2 1.92
4 2.44
Nt-cis-4
1 1.60
5 2.53
3 1.82
2 1.69
4 2.31
K-trans-10
2 1.78
4 2.16
3 1.72
5 2.01
6 2.74
1 1.69

S4. QTAIM Data for H+KPGG and Methylated Analogues

Hydrogen bonds numbered by strength and labeled in main text or section S3. All quantities in atomic units.

K-trans-1	ρ	$\nabla^2\rho$	V(r)
1	0.04803	0.14994	−0.04740
2	0.02455	0.09704	−0.01872
3	0.02007	0.08240	−0.01458

Nt-cis-1	ρ	$\nabla^2\rho$	V(r)
1	0.08092	0.07705	−0.08219
2	0.04188	0.14929	−0.04079
3	0.03215	0.12237	−0.02723

K-trans-2	ρ	$\nabla^2\rho$	V(r)
1	0.03364	0.12000	−0.02853
2	0.02810	0.10872	−0.02258
3	0.02031	0.07781	−0.01433
4	0.01779	0.05498	−0.01037
5	0.01068	0.04065	−0.00724
6	0.00877	0.02675	−0.00521

Nt-cis-2	ρ	$\nabla^2\rho$	V(r)
97	0.06969	0.08593	−0.06748
94	0.03952	0.14012	−0.03707
91	0.03342	0.12562	−0.02867
89	0.01358	0.04173	−0.00850
104	0.01242	0.04166	−0.00753

K-trans-3	ρ	$\nabla^2\rho$	V(r)
85	0.04294	0.14173	−0.04058
105	0.03929	0.13632	−0.03628
92	0.02529	0.10184	−0.01999
97	0.01284	0.04036	−0.00794
67	0.00878	0.02988	−0.00555

K-trans-4	ρ	$\nabla^2\rho$	V(r)
1	0.03656	0.13036	−0.03235
2	0.03149	0.11518	−0.02598
3	0.02563	0.10168	−0.01995

K-trans-5	ρ	$\nabla^2\rho$	V(r)
1	0.04366	0.14537	−0.04210
2	0.04301	0.14048	−0.04054
3	0.02359	0.09521	−0.01810
4	0.01082	0.03507	−0.00676
5	0.00726	0.02620	−0.00469

K-trans-6	ρ	$\nabla^2\rho$	V(r)
1	0.04597	0.14673	−0.04468
2	0.03334	0.11934	−0.02814
3	0.02276	0.09571	−0.01732
4	0.01767	0.07399	−0.01281
5	0.01750	0.06270	−0.01184
6	0.00868	0.02936	−0.00516

K-trans-7	ρ	$\nabla^2\rho$	V(r)
1	0.04293	0.14036	−0.04018
2	0.03566	0.12365	−0.03082
3	0.02239	0.09211	−0.01703
4	0.01210	0.04453	−0.00775
5	0.00735	0.02620	−0.00472

Nt-cis-3	ρ	$\nabla^2\rho$	V(r)
1	0.12205	0.04946	−0.15099
2	0.03894	0.14084	−0.03594
3	0.03926	0.13231	−0.03518
4	0.01352	0.04747	−0.00828
5	0.01022	0.03436	−0.00677
6	0.00915	0.02992	−0.00581
7	0.00782	0.02778	−0.00512

Nt-cis-4	ρ	$\nabla^2\rho$	V(r)
1	0.07004	0.08568	−0.06803
2	0.04289	0.14795	−0.04156
3	0.03341	0.12558	−0.02863
4	0.01271	0.04270	−0.00774
5	0.00942	0.02887	−0.00576

Me1-K-trans	ρ	$\nabla^2\rho$	V(r)
1	0.04178	0.13946	−0.03879
2	0.04051	0.13951	−0.03773
3	0.02440	0.09945	−0.01910
4	0.01266	0.04371	−0.00836
5	0.00924	0.03086	−0.00550

Me2-K-trans	ρ	$\nabla^2\rho$	V(r)
1	0.06186	0.14868	−0.06480
2	0.04781	0.13503	−0.04435
3	0.02796	0.11092	−0.02293
4	0.00907	0.03006	−0.00569
5	0.00790	0.02665	−0.00477

K-trans-8	ρ	$\nabla^2\rho$	V(r)
1	0.05646	0.14432	−0.05732
2	0.05299	0.09261	−0.04612
3	0.04497	0.13211	−0.04097
4	0.02733	0.11115	−0.02163
5	0.02411	0.08886	−0.01782
6	0.00913	0.02753	−0.00543

K-trans-9	ρ	$\nabla^2\rho$	V(r)
1	0.04847	0.14953	−0.04785
2	0.02566	0.09942	−0.01977
3	0.01809	0.05306	−0.01019
4	0.00964	0.03076	−0.00571

K-trans-10	ρ	$\nabla^2\rho$	V(r)
1	0.04651	0.13244	−0.04398
2	0.04737	0.09206	−0.03924
3	0.04119	0.14481	−0.03887
4	0.02082	0.09345	−0.01662
5	0.02086	0.08326	−0.01528
6	0.00760	0.02710	−0.00501

Me1-Nt-cis	ρ	$\nabla^2\rho$	V(r)
1	0.07932	0.14030	−0.08600
2	0.04565	0.14060	−0.04309
3	0.03916	0.13939	−0.03592
4	0.01347	0.04747	−0.00916
5	0.01112	0.03517	−0.00702

Me2-Nt-cis	ρ	$\nabla^2\rho$	V(r)
1	0.08002	0.14842	−0.08820
2	0.04632	0.14096	−0.04380
3	0.03710	0.13633	−0.03348
4	0.01001	0.03339	−0.00658
5	0.00916	0.03154	−0.00595
